# A Mix of Potentially Probiotic *Limosilactobacillus fermentum* Strains Alters the Gut Microbiota in a Dose- and Sex-Dependent Manner in Wistar Rats

**DOI:** 10.3390/microorganisms12040659

**Published:** 2024-03-26

**Authors:** Lucas Alves Carneiro dos Santos, Rodrigo Dias de Oliveira Carvalho, José Patrocínio Ribeiro Cruz Neto, Deborah Emanuelle de Albuquerque Lemos, Kataryne Árabe Rimá de Oliveira, Karoliny Brito Sampaio, Micaelle Oliveira de Luna Freire, Flavia Figueira Aburjaile, Vasco Ariston de Carvalho Azevedo, Evandro Leite de Souza, José Luiz de Brito Alves

**Affiliations:** 1 Department of Nutrition, Health Sciences Center, Federal University of Paraíba, João Pessoa 58051-900, Brazil; lucasalves.nutri@gmail.com (L.A.C.d.S.); patrocinio.neto.eltc@gmail.com (J.P.R.C.N.); deborah.albuquerque@academico.ufpb.br (D.E.d.A.L.); kataryne.arabe@academico.ufpb.br (K.Á.R.d.O.); karolbsampaio@gmail.com (K.B.S.); micamacario@gmail.com (M.O.d.L.F.); els@academico.ufpb.br (E.L.d.S.); 2 Department of Genetics, Ecology and Evolution, Federal University of Minas Gerais, Belo Horizonte 31270-901, Brazil; rodrigodoc2@gmail.com; 3 Department of Preventive Veterinary Medicine, Veterinary School, Federal University of Minas Gerais, Belo Horizonte 31270-901, Brazil; faburjaile@gmail.com (F.F.A.); vascoariston@gmail.com (V.A.d.C.A.)

**Keywords:** probiotics, *Limosilactobacillus*, gut microbiota, dose–response, sex differences

## Abstract

Multi-strain *Limosilactobacillus* (L.) *fermentum* is a potential probiotic with reported immunomodulatory properties. This study aimed to evaluate the composition, richness, and diversity of the gut microbiota in male and female rats after treatment with a multi-strain of *L. fermentum* at different doses. Thirty rats (fifteen male and fifteen female) were allocated into a control group (CTL), a group receiving *L. fermentum* at a dose of 10^8^ CFU (Lf-10^8^), and a group receiving *L. fermentum* at a dose of 10^10^ CFU (Lf-10^10^) for 13 weeks. Gut microbiota and serum cytokine levels were evaluated after *L. fermentum* treatment. Male CTL rats had a lower relative abundance of *Bifidobacteriaceae* and *Prevotella* and a lower alpha diversity than their female CTL counterparts (*p* < 0.05). In addition, male CTL rats had a higher *Firmicutes/Bacteroidetes* (F/B) ratio than female CTL rats (*p* < 0.05). In female rats, the administration of *L. fermentum* at 10^8^ CFU decreased the relative abundance of *Bifidobacteriaceae* and *Anaerobiospirillum* and increased *Lactobacillus* (*p* < 0.05). In male rats, the administration of *L. fermentum* at 10^10^ CFU decreased the F/B ratio and increased *Lachnospiraceae* and the diversity of the gut microbiota (*p* < 0.05). The relative abundance of *Lachnospiraceae* and the alpha-diversity of gut microbiota were negatively correlated with serum levels of IL1β (r = −0.44) and TNFα (r = −0.39), respectively. This study identified important changes in gut microbiota between male and female rats and showed that a lower dose of *L. fermentum* may have more beneficial effects on gut microbiota in females, while a higher dose may result in more beneficial effects on gut microbiota in male rats.

## 1. Introduction

Evidence from clinical and animal studies has shown that host sex influences the gut microbiota [1]. In healthy humans, the relative abundance of *Bacteroidetes* is typically lower in females than in males [2], whereas the relative abundance of the genus *Prevotella* is higher in males than in females [3]. In mice, the phyla *Actinobacteria* and *Tenericutes* were more abundant in males, while the family *Lachnospiraceae* was more abundant in females [4]. In addition, previous studies have shown that sex differences are associated with several diseases, including colorectal cancer [5], Parkison’s disease [6], essential hypertension [7], and ischemic stroke [8].

It is recognized that the administration of probiotics in adequate amounts can improve the composition, diversity, and function of the gut microbiota and promote host health benefits [9,10], such as in the treatment of cardiometabolic, cancer, inflammatory, and immune diseases [11,12,13,14]. The appropriate or effective dose of probiotics for overall health, gut microbiota, bowel function, and immune strength is a gap in the research [15]. Doses of around 1 × 10^9^ CFU/day (one billion CFU) have been used in studies to prevent or treat disrupted gut microbiota [16,17,18]. Given the interactions between the host and the microbiome, it has been suggested that gender may be a key aspect that can influence how probiotics may exert their effects on the gut microbiota in a host system [19].

A mixed-fruit-derived *Limosilactobacillus fermentum* developed by our research group has been reported as safe in a series of in vitro and in vivo experiments [20] and as having broad probiotic properties, such as the normalization of disturbed gut microbiota and antioxidant and anti-inflammatory properties when administered at 1 × 10^9^ CFU/day [21,22,23,24]. However, it is unclear whether mixed *L. fermentum* can modulate the gut microbiota in a dose- and sex-specific manner. Looking to develop a live biotherapeutic product that overcomes the major inconsistencies across studies with probiotic therapy, such as dose, duration of treatment, and male/female mixed population, the main endpoint of this study was to evaluate the dose- and sex-response of the gut microbiota in Wistar rats after the administration of a multi-strain mixture of *L. fermentum* 139, 263, and 296.

There is increasing evidence that the commensal gut microbiota can regulate local and systemic inflammation [15]. Therefore, we secondarily analyzed the correlation between inflammatory biomarkers and changes in the gut microbiota induced by probiotic administration to expand the available information and enrich the evidence on gut microbiota and inflammation after probiotic therapy.

## 2. Materials and Methods

### 2.1. Animals and Ethical Aspects

Thirty Wistar rats (fifteen male and fifteen female) were housed in polypropylene cages with filtered water and chow (Labina, Purina Aribands) ad libitum throughout the experiment, maintained on a 12 h light–dark cycle, with temperature of 22 ± 2 °C and controlled humidity (55 ± 10%). This study was approved by the Animal Experimentation Ethics Committee of the Federal University of Paraiba (CEUA/UFPB), under number 1871160322, and followed the recommendations of the National Council for the Control of Animal Experimentation (CONCEA, Sao Paulo, Brazil) and the International Principles for Biomedical Research Involving Animals.

### 2.2. Probiotic Strains and Reparation of Cell Suspension

The strains *L. fermentum* 139, *L. fermentum* 263, and *L. fermentum* 296 were kindly provided by the Laboratory of Microbiology, Department of Nutrition, Federal University of Paraíba (João Pessoa, PB, Brazil). Each strain was cultured anaerobically (Anaerobic System Anaerogen, Oxoid, Hampshire, UK) in Mann, Rogosa, and Sharpe (MRS) broth (Himedia, Mumbai, India) at 37 ± 0.5 °C for 20–24 h. To obtain the cell suspension, the cells were collected by centrifugation (8000× *g*, 10 min, 4 °C), washed twice with sterile PBS solution, resuspended in PBS solution, and homogenized by vortexing (30 s) to obtain standard cell suspensions with optical density (OD) at 625 nm (OD625) of 1.2 and 2.0, corresponding to viable cell counts of approximately 10^8^ colony-forming units per milliliter (CFU/mL) and 10^10^ CFU/mL, respectively, when plated on MRS agar (HiMedia, Thane, India). In order to increase the specific strain characteristics and to obtain a multi-strain probiotic, mixed cell suspensions were prepared at a ratio of 1:1:1 (*v*/*v*). These doses have been tested to achieve 1 log below and 1 log above a dose widely considered therapeutic (10^9^ CFU).

### 2.3. Experimental Design

Male and female rats were grouped into (i) control group (CTL, n = 5/sex), (ii) *L. fermentum* receiving a dose of 10^8^ CFU (Lf-10^8^, n = 5/sex), and (iii) *L. fermentum* receiving a dose of 10^10^ CFU (Lf-10^10^, n = 5/sex). The control group received PBS as a placebo vehicle. Placebo or *L. fermentum* was administered by oral gavage at a dose of 1 mL for 13 weeks. Then, 24 h after the last dose of *L. fermentum*, blood samples were collected for cytokine analysis and feces were collected for gut microbiota analysis.

### 2.4. Measurement of Cytokines

At the end of the experimental test, all animals were euthanized with an overdose of anesthetic. Blood samples were collected and centrifuged to separate serum and plasma. Serum samples were stored at −80 °C in a freezer until the time of cytokine analysis.

Cytokine levels in blood serum samples (TNF-α, IL-1β, IL-6, and IL-10) were determined using the Millipore 7-plex kit (Millipore Corp., Billerica, MA, USA). The assay was performed in a 96-well plate with a filter membrane according to the manufacturer’s instructions. Cytokine concentrations in the samples were estimated from a standard curve using a third-order polynomial equation and expressed in pg/mL. Samples below the limit of detection of the assay were recorded as zero, while samples above the highest quantification limit of the standard curve were assigned to the highest value on the curve. Reading was performed using a microplate reader [21].

### 2.5. DNA Extraction, 16S rRNA Gene Amplicon Library Preparation, and Sequencing

Fecal samples were collected directly from the animals’ colons and stored in a −80 °C freezer for later analysis. Total DNA was extracted using the QIAmp PowerFceal^®^ DNA Kit, and a region of approximately 426 bp encompassing the V3 and V4 hypervariable regions of the 16S rDNA gene was targeted for the sequencing of each sample.

The bacterial diversity was assessed via the high-throughput sequencing of the 16S rRNA V3/V4 region employing 341F (CCTACGGGRSGCAGCA G) and 806R (GGACTACHVGGGTWTCTAAT) primers. The 16S rRNA libraries were sequenced using the MiSeq Sequencing System (Illumina Inc., San Diego, CA, USA) using the standard Illumina primers provided in the kit, with 300 cycles (paired-end sequencing with 200 bp). After sequencing, quality filters were applied to fastq files, including for the removal of truncated and low-quality reads (Phred score < 20) using the Trimmomatic tool [25]. Then, sense and antisense paired reads were merged into contigs, and the singletons and chimeras were removed. The sequences were grouped into Taxonomic Operational Units (OTUs) using Uchime v. 4.2.40 and Vsearch v 2.22.1 [26,27] (97% identity) and assigned taxonomically considering a 97% similarity alignment against sequences from the SILVA database [28]. All 16 s rRNA Illumina amplicon sequencing data provided in this study can be publicly obtained from the Sequence Read Archive (SRA) of The National Center for Biotechnology Information (NCBI) under the accession number PRJNA1004239.

### 2.6. Statistical Analysis

Data are presented as mean ± standard deviation. The Shapiro–Wilk test was used to assess the normality of the data. Statistical significance was assessed using a two-way analysis of variance ANOVA test with dose (10^8^ and 10^10^ CFU) and sex (male and female) as factors. The Bonferroni post hoc test was used. Pearson’s correlation test was used. Statistical analysis was performed using GraphPad Prism^®^ (version 6.01) and significance was maintained at *p* < 0.05. Data were analyzed with GraphPad Prism 8.0 (GraphPad Software, San Diego, CA, USA).

## 3. Results

### 3.1. Effects of Multi-Strain L. fermentum Administration on Bacterial Phyla Composition in Gut Microbiota

**Figure 1:**
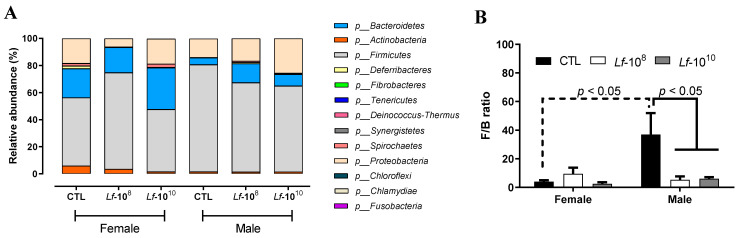
Relative abundance of phylum (**A**) and *Firmicutes/Bacteroides* ratio (**B**) in the gut microbiota of male and female *Wistar* rats after the administration of *Limosilactobacillus fermentum* 139, 263, and 296 at different doses for 13 weeks. F/B ratio data are presented as mean ± standard deviation and analyzed using two-way ANOVA. *p* < 0.05 indicates a significant difference. A dotted line was used to identify significant differences between sex and a solid line was used to identify significant differences in *L. fermentum* administration. Groups: control group (CTL): PBS (1 mL), *L. fermentum* receiving a dose of 10^8^ CFU/mL (Lf-10^8^), and *L. fermentum* receiving a dose of 10^10^ CFU/mL (Lf-10^10^).

Thirteen phyla were identified by 16S rRNA sequencing (Figure 1A). The most abundant phyla detected were *Firmicutes*, *Bacteroidetes*, and *Proteobacteria*, followed by *Actinobacteria* (Figure 1A). Female rats of the CTL group had a reduced *Firmicutes/Bacteroidetes* ratio compared to male CTL rats (*p* < 0.05, Figure 1B). The administration of *L. fermentum* at 10^8^ and 10^10^ CFU doses did not change the *Firmicutes/Bacteroidetes* ratio in female rats, but significantly reduced the *Firmicutes/Bacteroidetes* ratio in male rats (*p* < 0.05, Figure 1B).

### 3.2. Effects of Multi-Strain L. fermentum Administration on Bacterial Family Composition in Gut Microbiota

**Figure 2:**
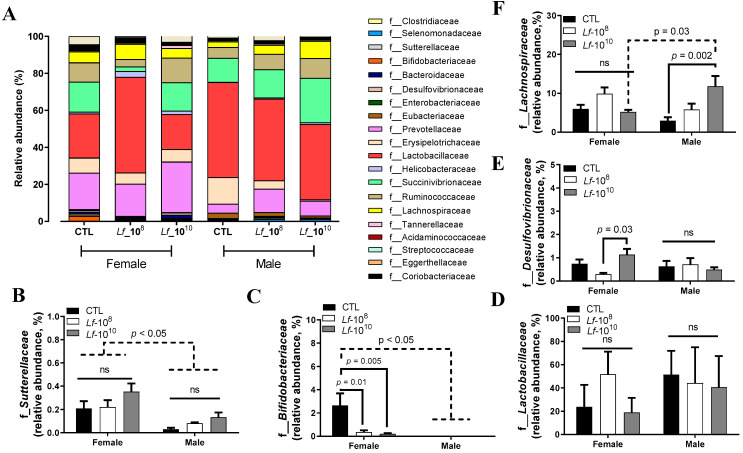
Relative abundance of bacterial families in the gut microbiota of male and female Wistar rats after the administration of *Limosilactobacillus fermentum* 139, 263, and 296 at different doses for 13 weeks. Evaluation of the most abundant families identified in the gut microbiota of male and female rats (**A**). Relative abundances of *Sutterellaceae* (**B**), *Bifidobacteriaceae* (**C**), *Lactobacillaceae* (**D**), *Desulfovibrionaceae* (**E**), and *Lachnospiraceae* (**F**) were analyzed by two-way ANOVA. *p* < 0.05 indicates a significant difference. A dotted line was used to identify significant difference between sex and a solid line was used to identify significant difference in *L. fermentum* administration. Groups: control group (CTL): PBS (1 mL), *L. fermentum* receiving a dose of 10^8^ CFU/mL (Lf-10^8^), and *L. fermentum* receiving a dose of 10^10^ CFU/mL (Lf-10^10^). Not significant: ns.

The most abundant phyla detected were *Clostridiaceae*, *Selenomonadaceae*, *Sutterellaceae*, *Bifidobacteriaceae*, *Bacteroidaceae*, *Desulfovibrionaceae*, *Enterobacteriaceae*, *Eubacteriaceae*, *Prevotellaceae*, *Erysipelotrichaceae*, *Lactobacillaceae*, *Helicobacteraceae*, *Succinivibrionaceae*, *Ruminococcaceae*, *Lachnospiraceae*, *Tannerellaceae*, *Acidaminococcaceae*, *Streptococcaceae*, *Eggerthellaceae*, and *Corlobacteriacea* (Figure 2A).

Female rats of all groups had a higher abundance of *Sutterellaceae* and *Bifidobacteriaceae* when compared to male rats (*p* < 0.05, Figure 2B,C). The administration of *L. fermentum* at 10^8^ or 10^10^ CFU did not alter the abundance of *Sutterellaceae* in male and female rats (*p* > 0.05, Figure 2B). The administration of *L. fermentum* at 10^8^ or 10^10^ CFU reduced the relative abundance of *Bifidobacteriaceae* in female rats (*p* < 0.05, Figure 2C), but did not change that of *Bifidobacteriaceae* in male rats (*p* > 0.05, Figure 2C). The relative abundance of *Lactobacillaceae* was similar between female and male rats and the administration of *L. fermenutm* at 10^8^ or 10^10^ did not alter the relative abundance of *Lactobacillaceae* in male and female rats (*p* > 0.05, Figure 2D). The relative abundance of *Desulfovibrionaceae* was similar between female and male rats (*p* > 0.05, Figure 2E). Female rats receiving *L. fermentum* at 10^10^ CFU had a higher relative abundance of *Desulfovibrionaceae* than female rats receiving 10^8^ CFU (*p* < 0.05, Figure 2E). The administration of *L. fermentum* at 10^8^ or 10^10^ CFU did not alter the relative abundance of *Desulfovibrionaceae* in male rats (*p* > 0.05, Figure 2E). The administration of *L. fermentum* at 10^8^ or 10^10^ CFU did not alter the relative abundance of *Lachnospiraceae* in female rats (*p* > 0.05, Figure 2F). Male rats receiving *L. fermentum* at 10^10^ CFU had a higher relative abundance of *Lachnospiraceae* than male CTL rats (*p* < 0.05, Figure 2F). Male rats receiving *L. fermentum* at 10^10^ CFU had a higher relative abundance of *Lachnospiraceae* than female rats receiving the same dose (*p*< 0.05, Figure 2F).

### 3.3. Effects of Multi-Strain L. fermentum Administration on Bacterial Gender Composition in Gut Microbiota

**Figure 3:**
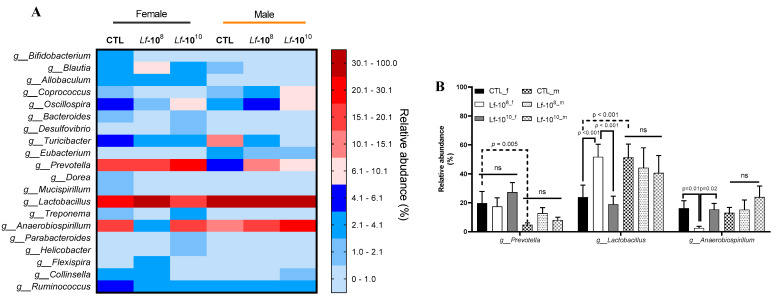
Heat map of the most abundant bacterial genera in male and female *Wistar* rats after administration of *Limosilactobacillus fermentum* 139, 263, and 296 at different doses for 13 weeks. Evaluation of the heat map of the most abundant genera (**A**) in the gut microbiota of male and female rats. Relative abundances of *Prevotella*, *Lactobacillus*, and *Anaerobiospirillum* (**B**) were analyzed by two-way ANOVA. *p* < 0.05 indicates a significant difference. A dotted line was used to identify significant difference between sex and a solid line was used to identify significant difference in *L. fermentum* administration. Groups: control group (CTL): PBS (1 mL), *L. fermentum* receiving a dose of 10^8^ CFU/mL (Lf-10^8^), and *L. fermentum* receiving a dose of 10^10^ CFU/mL (Lf-10^10^). not significant (ns).

The most abundant genera detected in the gut microbiota are shown in Figure 3A. The genera *Lactobacillus*, *Prevotella*, and *Anaerobiospirillum* showed higher relative abundance in female and male rats (Figure 3A). The female CTL group had a higher relative abundance of *Prevotella* than the male CTL group (*p* < 0.05, Figure 3B). The administration of *L. fermentum* at 10^8^ or 10^10^ CFU did not alter the relative abundance of *Prevotella* in female and male rats (*p* > 0.05, Figure 3B). The female CTL group had a lower relative abundance of *Lactobacillus* than the male CTL group (*p* < 0.05, Figure 3B). The administration of *L. fermentum* at 10^8^ or 10^10^ CFU did not alter the relative abundance of *Lactobacillus* in male rats (*p* > 0.05, Figure 3B). In female rats, the administration of *L. fermentum* at 10^8^ CFU increased the relative abundance of *Lactobacillus* when compared to the CTL group. However, the administration of *L. fermentum* at 10^10^ CFU promoted deleterious effects on *Lactobacillus* abundance when compared to female rats receiving *L. fermentum* at 10^8^ CFU (*p* < 0.05, Figure 3B). The relative abundance of *Anaerobiospirillum* was similar between female and male rats (*p* > 0.05, Figure 3B). The administration of *L. fermentum* at 10^8^ or 10^10^ CFU did not alter the relative abundance of *Anaerobiospirillum* in male rats (*p* > 0.05, Figure 3B). In female rats, the administration of *L. fermentum* at 10^8^ CFU decreased the relative abundance of *Anaerobiospirillum* when compared to the CTL group (*p* < 0.05, Figure 3B). The administration of *L. fermentum* at 10^10^ CFU increased the abundance of *Anaerobiospirillum* when compared to female rats receiving *L. fermentum* at 10^8^ CFU (*p* < 0.05, Figure 3B).

### 3.4. Effects of Multi-Strain L. fermentum Administration on the Richness and Diversity of the Gut Microbiota

**Figure 4:**
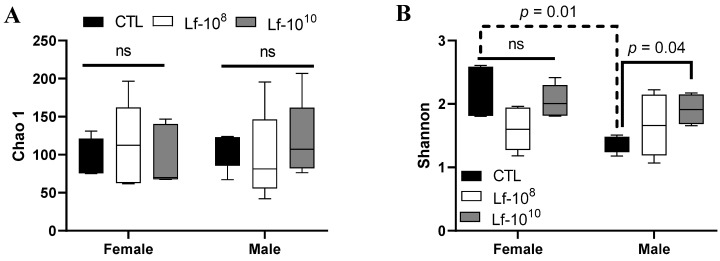
Richness and alpha diversity of male and female Wistar rats after administration of *Limosilactobacillus fermentum* 139, 263, and 296 at different doses for 13 weeks. Evaluation of the Chao 1 index (**A**) and Shannon index (**B**) in in the gut microbiota of male and female rats. Data were analyzed by two-way ANOVA. *P* < 0.05 indicates a significant difference. A dotted line was used to identify significant difference between sex and a solid line was used to identify significant difference in *L. fermentum* administration. Groups: control group (CTL): PBS (1 mL), *L. fermentum* receiving a dose of 10^8^ CFU/mL (Lf-10^8^), and *L. fermentum* receiving a dose of 10^10^ CFU/mL (Lf-10^10^). not significant (ns).

Species richness was estimated using the Chao 1 index and the alpha diversity was assessed using the Shannon index (Figure 4A,B). Species richness was similar between male and female rats, and the administration of *L. fementum* did not alter the Chao 1 index in either sex (*p* > 0.05, Figure 4A). Male rats of the CTL group had lower alpha diversity than their counterpart CTL female rats (*p* < 0.05, Figure 4B). The administration of *L. fermentum* at 10^10^ CFU increased alpha diversity in male rats compared to the CTL group (*p* < 0.05, Figure 4B), but had no effect in female rats (*p* > 0.05, Figure 4B).

### 3.5. Correlation between the Gut Microbiota Parameters of Inflammatory Cytokines

**Figure 5:**
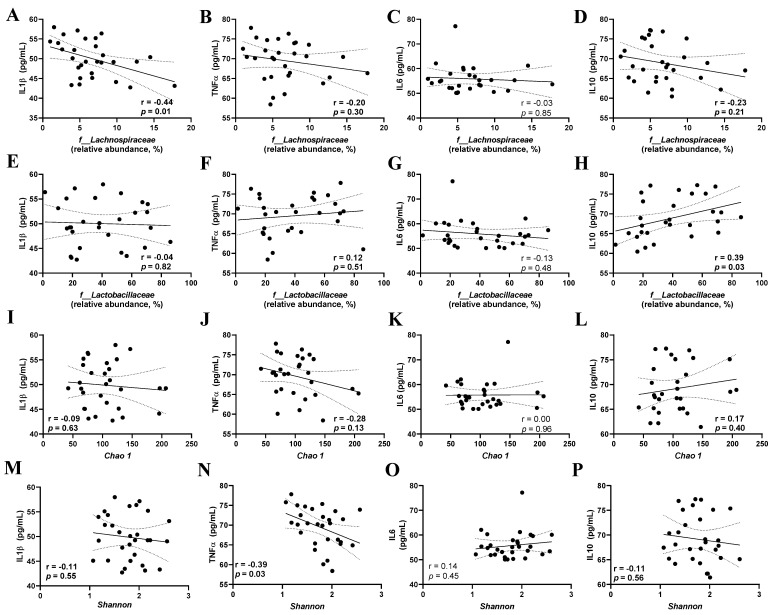
Correlation between cytokine serum levels and gut microbiota parameters in male and female *Wistar* rats after the administration of *Limosilactobacillus fermentum* 139, 263, and 296 at different doses for 13 weeks. Evaluation of the correlation of *Lachnospiraceae* (**A**–**D**), *Lactobacillaceae* (**E**–**H**), Chao 1 Index (**I**–**L**), and Shannon Index (**M**–**P**) with serum levels of Interleukin 1 beta (IL1β), Tumor Necrosis Factor alpha (TNF-α), Interleukin-6 (IL–6), and Interleukin-10 (IL–10). Pearson’s correlation test was used, and significant difference was considered when *p* < 0.05.

The relative abundance of *Lachnospiraceae* and *Lactobacillaceae* and the Chao 1 and Shannon indices were used to assess the correlation between gut microbiota parameters and inflammatory cytokines. The relative abundance of *Lachnospiraceae* was negatively correlated with IL-1β levels (r = 0.44, *p* = 0.01, Figure 5A), but not with TNF-α, IL-6, and IL-10 levels (*p* > 0.05, Figure 5B–D). The relative abundance of *Lactobacillaceae* was positively correlated with IL-10 levels (r = 0.39, *p* = 0.03, Figure 5H), but not with IL-1β, TNF-α, and IL-6 levels (*p* > 0.05, Figure 5E–G). The Chao 1 index did not correlate with IL-1β, TNF-α, IL-6, and IL-10 levels (*p* > 0.05, Figure 5I–L). The Shannon index was negatively correlated with TNF-α levels (*p* < 0.05, Figure 5N), but not with IL1β, IL-6, and IL-10 levels (*p* > 0.05, Figure 5M,O,P).

## 4. Discussion

This study showed changes in the gut microbiota composition of male and female Wistar rats after treatment with *L. fermentum* 139, 263, and 296 at different doses for 13 weeks. The results showed that multi-strain *L. fermentum* treatment altered the relative abundance of bacteria at the phylum, family, and genus levels. The relative abundance of *Lachnospiraceae* was negatively associated with serum IL1β levels, while the relative abundance of *Lactobacillaceae* was positively associated with serum IL-10 levels. In addition, alpha diversity was negatively correlated with TNFα. It has been suggested that the composition of the gut microbiota is sex-dependent [1,29] and may respond differently to probiotic treatment [19,30] and that lactobacilli can alter the population of microorganisms that make up the gut microbiota and control the functioning of the gut microbiota ecosystem [10].

The most abundant bacterial phyla in the healthy gut microbiota are represented by *Firmicutes* and *Bacteroidetes* [31]. The *Firmicutes*/*Bacteroidetes* ratio has been used as a potential biomarker for obesity and associated disorders [32]. An increased *Firmicutes*/*Bacteroidetes* ratio has been reported in several diseases, such as obesity, diabetes mellitus, inflammatory bowel disease, and cardiovascular disease [33,34,35]. On the other hand, a low *Firmicutes/Bacteroidetes* ratio has been associated with a lean phenotype, younger age, cardiovascular health, and an improved immune system [32]. Treatment with Lf-10^8^ or Lf-10^10^ decreased the *Firmicutes/Bacteroidetes* ratio in male rats compared to the CTL group, while no change was observed in female rats. These results suggest that treatment with *L. fermentum* 139, 263, and 296 can positively modulate the gut microbiota composition at the phyla level.

*Lactobacillaceae* was the family with the highest relative abundance in the gut microbiota of Wistar rats. The *Lactobacillaceae* family can be found in different environments, such as the gastrointestinal tract and urinary and genital systems [34]. Although the treatment with *L. fermentum* 139, 263, and 296 did not alter the relative abundance of *Lactobacillaceae* in male and female rats, many *Lactobacillus* species are used as probiotics due to strain-specific properties, such as cholesterol-lowering activity, immunomodulatory effects, and antioxidant properties [36,37,38,39].

*Lachnospiraceae* is a family of anaerobic bacteria in the *Clostridiales* order within the phylum *Firmicutes*, and they are obligate members of the gut microbiota in healthy humans [40]. An increased abundance in short-chain fatty acid (SCFA)-producing bacteria belonging to the *Lachnospiraceae* family has been reported in subjects fed a high-fiber diet or treated with omega-3 polyunsaturated fatty acids (PUFA), and has been associated with host health benefits [41]. On the other hand, *Lachnospiraceae*-enriched gut microbiota have been reported in patients with chronic and inflammatory diseases [42]. Treatment with *L. fermentum* 139, 263, and 296 increased the relative abundance of *Lachnospiraceae* in male rats receiving Lf-10^10^ when compared to the CTL group. However, the treatment with *L. fermentum* 139, 263, and 296 did not change the relative abundance of *Lachnospiraceae* in female rats. The reason for this is not explained and reinforces the idea that probiotic therapy may have a sex-specific effect on the gut microbiota.

*Bifidobacteriaceae* are a family of bacteria with fermentative metabolism that inhabit the gastrointestinal tract of humans and animals [43]. A previous meta-analysis suggested that high populations of *Bifidobacteriaceae* may be involved in the pathogenesis of Parkinson’s disease [44], while a systematic review indicated a higher abundance of *Bifidobacteriaceae* in individuals with depression [45]. Our results showed a decrease in the relative abundance of *Bifidobacteriaceae* in male rats when compared with female rats. In addition, the treatment with Lf-10^8^ and Lf-10^10^ decreased the relative abundance of *Bifidobacteriaceae* in female rats when compared to their CTL group.

A preclinical study showed that the consumption of ground beef and sucrose stimulated an expansion of the *Desulfovibrionaceae* family in the colonic microbiome, which was associated with oxidative stress and cardiac hypertrophy [46]. High-fat diet consumption increased the relative abundance of *Desulfovibrionaceae* in mice [47]. In the present study, the administration of Lf-10^10^ increased the relative abundance of the *Desulfovibrionaceae* family in female rats compared to the dose of 10^8^ CFU/mL and the CTL group, although no difference was found when compared to male rats.

It has been shown that the relative abundance of *Sutterellaceae* was increased in fecal samples from patients with irritable bowel syndrome [48]. In the present study, the relative abundance of *Sutterellaceae* was lower in male rats when compared with female rats. Further studies may be conducted to determine whether females have a higher risk of developing irritable bowel syndrome. The administration of *L. fermentum* did not alter the relative abundance of *Sutterellaceae* in either sex. It has been demonstrated that oats, a soluble fiber used as a prebiotic, decreased the relative abundance of *Sutterellaceae* in a Chinese population with mild hypercholesterolemia [49]. In addition, the abundance of *Sutterellaceae* was negatively correlated with quercetin concentration in healthy elderly humans [50]. Our research group has developed a novel nutraceutical product containing prebiotics, polyphenols, and *L. fermentum* strains [51,52], and further studies will be conducted to understand their effects on gut microbiota composition in health and disease.

The composition of the gut microbiota was also assessed at the genus level. In the present study, the genera with the highest relative abundance were *Lactobacillus*, *Prevotella*, and *Anaerobiospirillum*. The genus *Anaerobiospirillum* is understudied in the literature. An early study showed *Anaerobiospirillum succiniproducens*-induced bacteremia in a healthy man [53]. Here, we have shown that the administration of *L. fermentum* at 10^8^ CFU decreased the relative abundance of *Anaerobiospirillum* in female rats, but such an effect was absent when 10^10^ CFU of *L. fermentum* was administered.

Increased *Lactobacillus* counts in the feces of rats treated with *L. fermentum* strains have previously been documented [23,39]. This study showed that females have a lower relative abundance of *Lactobacillus* than males, and the administration of *L. fermentum* at 10^8^ CFU may be more beneficial to *Lactobacillus* abundance in females than 10^10^ CFU. In males, *L. fermentum* treatment did not alter the relative abundance of *Lactobacillus*. The benefits of *Lactobacillus* when used as a probiotic have been associated with improvements in metabolic, immunological, and cardiovascular parameters and may be a promising alternative for the management of inflammatory bowel diseases and cardiometabolic disorders [54,55,56].

*Prevotella* was one of the genera with the highest increase due to our treatment with *L. fermentum*. Females have a higher relative abundance of *Prevotella* than males, and the administration of *L. fermentum* did not alter *Prevotella* abundance in either sex. This genus belongs to the family *Prevotellaceae*, and compared to other genera, *Prevotella* has received less attention [57]. *Prevotella* species can have different characteristics between and within species, but their functions and host relationships are still unclear [58]. Although the abundance of this bacterial genus is evident in the healthy microbiota, studies have suggested that some members may be associated with diseases, including bacterial vaginosis and inflammatory autoimmune diseases, but the direct causes are still uncertain [59,60]. There are conflicting reports in the literature regarding the effects of the genus *Prevotella* on glucose homeostasis [57,61]. A previous study using germ-free mice transplanted with microbiota from human donors and subjected to the consumption of barley-based bread showed an improvement in glucose metabolism associated with greater *Prevotella* abundance, possibly related to increased hepatic glycogen storage [62].

Probiotics are generally effective at oral doses greater than 10^6^ CFU, but the most commonly used doses in experiments are 10^8^ to 10^10^ CFU/mL [63]. Regarding dose–response, it was demonstrated in female rats that treatment with *L. fermentum* 139, 263, and 296 promoted a greater relative abundance of the *Lactobacillus* genus in the Lf-10^8^ group compared to the Lf-10^10^ groups. A previous study found that the dose–response effects of *Bifidobacterium infantilum* 35624 were effective in reducing irritable bowel syndrome in adult women at the 10^8^ CFU/day dose, with no significant difference between the 10^6^ and 10^10^ CFU/day doses and placebo [64]. The combination of these results suggests that identifying an appropriate dose is very important for probiotic therapy and that further studies should be conducted to evaluate the dose–response effects of potential probiotics.

Another critical parameter evaluated in this study was the effect of the administration of *L. fermentum* 139, 263, and 296 on the richness and alpha diversity of the gut microbiota in male and female rats. Increased microbial diversity has been associated with improved microbiota stability, with implications for host health benefits [65]. The study showed that although richness was similar between male and female rats, alpha diversity was higher in female rats than in male rats. The administration of *L. fermentum* did not change the species richness in either sex. Treatment with the higher dose of *L. fermentum* 139, 263, and 296 increased the alpha diversity of the gut microbiota in male rats. A previous study showed that the administration of *L. fermentum* did not increase alpha diversity in rats fed a diet high in fat and cholesterol [55]. Reduced gut microbiota diversity may be associated with clinical conditions such as obesity and inflammatory bowel disease [66,67]. These findings suggest that the response of gut microbiota diversity to probiotic treatment may vary depending on the health status of the host.

Physiologically, the gut microbiota is directly linked to the immune system in maintaining homeostasis in the host gut [68]. In addition, it has been suggested that the consumption of specific probiotic strains modulates the immune response via the innate and adaptive immune systems, the regulation of intestinal epithelial permeability, mucus secretion, and competition within the bacterial ecosystem via the secretion of antimicrobial compounds [69]. However, the mechanistic effects of *L. fermentum* 139, 263, and 296 on the host immune system remain to be elucidated. This study showed that the relative abundance of *Lachnospiraceae* was negatively correlated with IL-1β levels, the relative abundance of *Lactobacillaceae* was positively correlated with IL-10 levels, and the alpha diversity of the gut microbiota was negatively correlated with TNF-α levels.

The health-promoting effects of members of the *Lachnospiraceae* and *Lactobacillaceae* families have been described in the literature, including the production of SCFA, the conversion of primary bile acids into secondary bile acids, and the protection of the intestinal barrier by resisting the colonization of drug-resistant pathogens [40,42,70]. Another characteristic associated with members of these families is the modulation of the immune system [71]. The relationship between *Lachnospiraceae* and the immune system has previously been described, showing that colonization with *Lachnospiraceae* in mdr2 −/− mice pre-treated with antibiotics caused a reduction in liver fibrosis, inflammation, and pathobiont translocation, which could be mediated by *Lachnospiraceae* metabolites, such as SCFA [72].

The positive correlation between the relative abundance of *Lactobacillaceae* and IL-10 levels suggests that supplementation with these potentially probiotic strains may be associated with a beneficial modulation of the immune response. Species of the *Lactobacillaceae* family have been used as probiotics to improve human or animal health [15], including for anti-inflammatory properties [73]. The effects of probiotics on the immune system are mainly explained by their ability to increase SCFA production [74]. Among the SCFA, butyrate has typically been associated with these anti-inflammatory effects, in addition to providing energy to colonic epithelial cells and regulating the expression of intestinal barrier junction proteins [75].

The main limitation of this study is the lack of hormonal parameters that could be used to explain some changes related to probiotic therapy in male and female rats. We also pointed out as a limitation of the study that the environment in which the animals were housed did not correspond to the Specific Pathogen Free (SPF) barrier environment.

This study identified important changes in gut microbiota between male and female rats and showed that a lower dose of *L. fermentum* may have more beneficial effects on the gut microbiota in females, while a higher dose may result in more beneficial effects on the gut microbiota in males. The study showed that a higher relative abundance of *Lachnospiraceae* and gut microbiota diversity was negatively correlated with pro-inflammatory cytokines, while a higher relative abundance of *Lactobacillaceae* was positively correlated with serum levels of anti-inflammatory cytokines. Despite the evidence indicating these strains as novel candidates for probiotic use, there is a need to confirm their health benefits through a translational approach by developing randomized, double-blind, placebo-controlled trials to investigate their health-promoting effects in humans [76].

## Data Availability

The datasets generated and/or analyzed during the current study are available from the corresponding author upon reasonable request.
